# An unusual case report of a subhepatic appendix and an interlobar hepatic bridge in a patient with acute cholecystitis

**DOI:** 10.1093/jscr/rjad185

**Published:** 2023-04-12

**Authors:** Carissa McGuin, Yagan Pillay

**Affiliations:** College of Medicine, University of Saskatchewan, Saskatoon, Canada; Department of Surgery, University of Saskatchewan, Saskatoon, Canada

**Keywords:** Subhepatic appendix, Interlobar bridge, Hepatic bridge

## Abstract

A subhepatic appendix and an interlobar hepatic bridge are both rare anatomical variants. To find both entities in the same patient at the time of a laparoscopic cholecystectomy makes for a unique case report. Subhepatic appendicitis has a reported incidence of 0.08%, and there have been only published three case reports of an interlobar hepatic bridge. Their lack of involvement in acute cholecystitis facilitated an easier dissection process and prevented serious complications to the liver or the right hemi-colon.

## INTRODUCTION

A subhepatic appendix and an interlobar hepatic parenchymal bridge are rare anatomical entities. A case report with both conditions in the same patient makes for a truly unique presentation.

Their lack of involvement in the acute cholecystitis facilitated an easier laparoscopic gallbladder extraction and reduced the risk of more extensive complications to either the liver or the right hemi colon in this case report.

## CASE REPORT

A 54-year-old female patient was referred to the acute care surgical team with acute onset abdominal pain over the epigastrium. The pain radiated to her back, and there was a food–pain association with fatty meals. This was her second admission in a month, and she was previously treated for gallstone pancreatitis. Her pain profile was similar over the two hospital admissions. Clinically, her abdomen was tender on deep palpation over the epigastrium. She had no overt signs of peritonism. She remained hemodynamically stable throughout her clinical examination. Significant laboratory investigations included a leucocytosis and an elevated lipase over five times the normal range. Liver functions were normal.

Computerized tomography scan of the abdomen showed an acute cholecystitis as well as radiological features of an acute pancreatitis. There was no pancreatic necrosis, abscess or choledocholithiasis.

She was managed conservatively with analgesia, diet modification and antibiotics and responded well to medical management. After her clinical symptoms settled, we discussed further management, and she signed an informed consent for a laparoscopic cholecystectomy at this admission.

**Figure 1:**
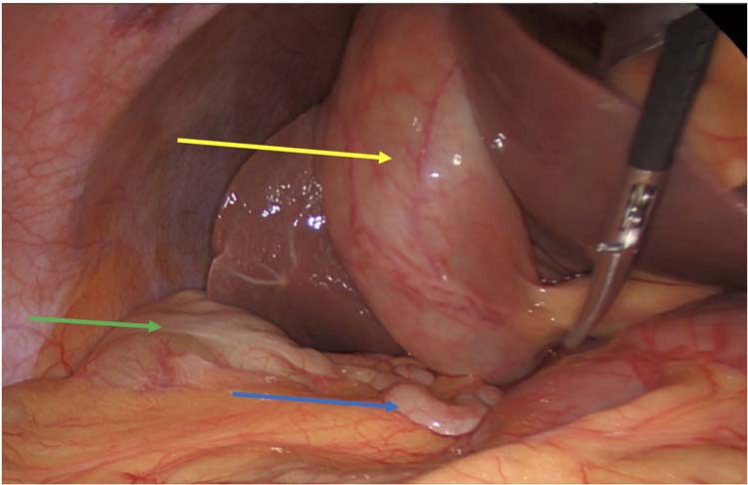
Acute cholecystitis (yellow arrow) with the high riding cecum (green arrow) and subhepatic appendix (blue arrow).

**Figure 2:**
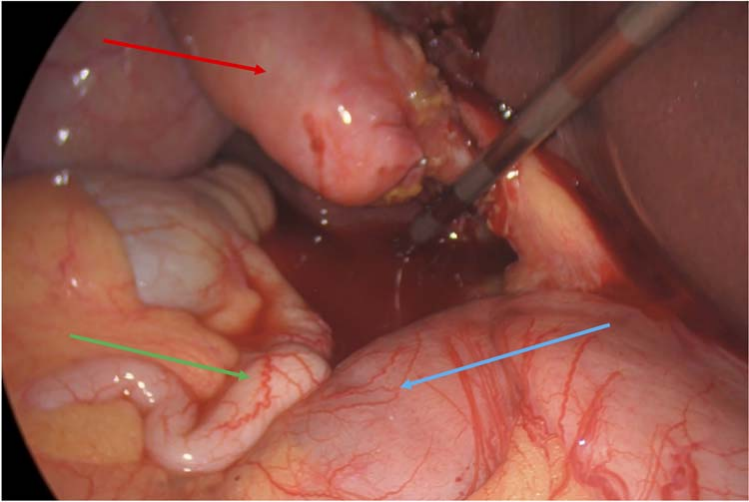
Subhepatic appendix (green arrow) in relation to the duodenum (blue arrow) and gallbladder (red arrow).

Intraoperatively she had severe inflammation at the Calot’s triangle, and we performed a retrograde, fundus down and cholecystectomy. She was noted to have a subhepatic appendix along with a high riding cecum (Figs 1 and 2). The appendix was not involved in the gallbladder inflammation (Fig. 4).

**Figure 3:**
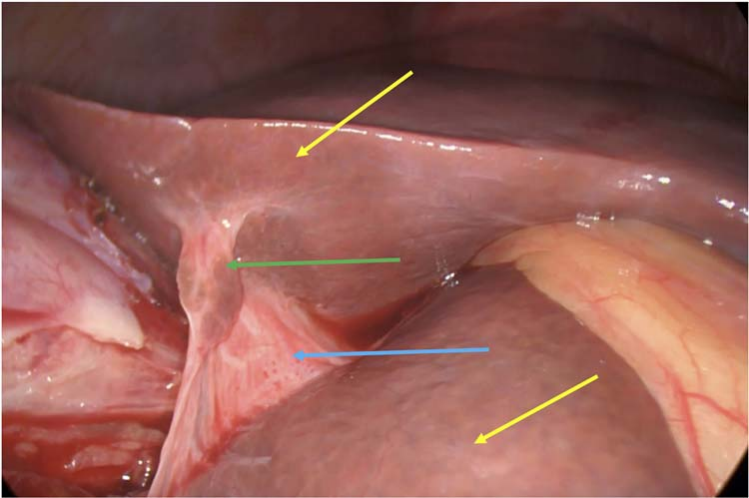
Incidental finding of a subhepatic bridge (blue arrow) with liver parenchymal tissue (green arrow) joining the left and right lobes of the liver (yellow arrows).

**Figure 4:**
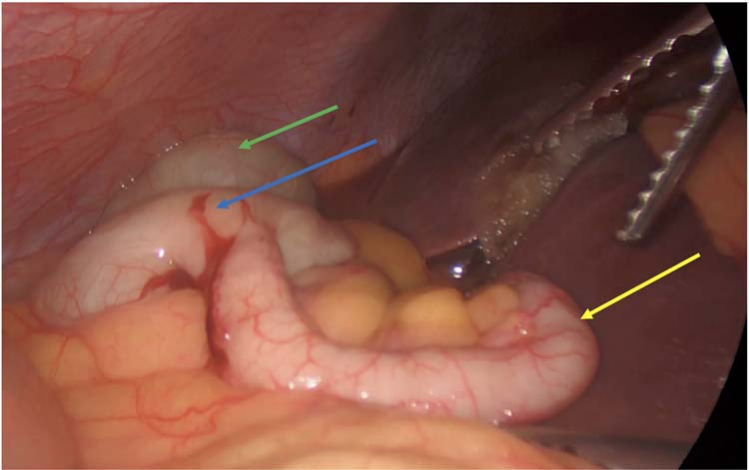
Post cholecystectomy with the subhepatic appendix overlying the left lobe of the liver (yellow arrow) and the cecum (blue arrow) next to the hepatic flexure (green arrow).

**Figure 5:**
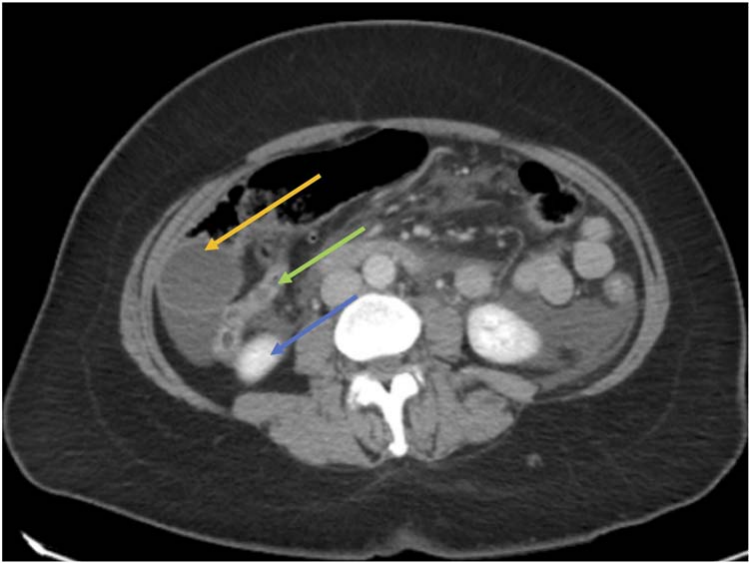
CT axial view showing the appendix (green arrow) inferior to the gallbladder (orange arrow) and superior to the right kidney (blue arrow).

Concurrently, we identified an interlobar hepatic bridge between the right and left liver lobes isolated from the cholecystitis (Fig. 3). The interlobar bridge contained hepatic tissue as well.

Her postoperative recovery was uneventful, and she was discharged home.

Pathology confirmed an acute cholecystitis.

## DISCUSSION

Subhepatic appendicitis was first described by King *et al.* in 1955 [1]. There have been a handful of cases presented in the published literature. Clinicians face a diagnostic difficulty in these cases due to the anatomical variation within the abdomen [2–4]. Delays in proper diagnosis can lead to complications such as appendiceal rupture and sepsis. The ubiquitous use of computerized tomography scans in patient’s radiological assessments helps to prevent such potential complications. Clinicians must be aware of the possible anatomical locational variants of the vermiform appendix. In most published series, the appendix is retrocecal (74%). The second commonest location is pelvic (21%), followed by subcecal, pre-ileal and post-ileal (1.5, 1 and 0.5%, respectively) [5]. An extensive review of 7210 cases of appendicitis found the incidence of subhepatic appendicitis to be 0.08% [6]. Incidental findings of a normal subhepatic appendix are extremely rare with only two such cases in the literature [7, 8] excluding our own. The lack of appendicular or cecal involvement facilitated an easier dissection of the gallbladder without a concomitant appendicectomy. Their involvement in the disease process could have increased the complexity of the surgery and possible complications. The presence of a high riding cecum and subhepatic appendix should arouse the clinician’s suspicion for an intestinal malrotation. Fortunately, in this case, the duodenum remains to the left of the cecum and hepatic flexure. A complete malrotation could have resulted in ischemia due to a possible vascular compromise of the superior mesenteric vessels.

Contributing to the unusual nature of this case was the presence of a subhepatic bridge containing liver parenchymal tissue. Only three other cases of an interlobar parenchymal bridge could be found in the literature [9–11]. The first was a cadaveric dissection with a variation in hepatic blood supply. Such anatomic variations are important to identify so that safe surgical techniques can be developed to avoid unnecessary blood loss and hepatic injury in these patients undergoing abdominal surgery. In the second case, the parenchymal bridge caused an internal hernia that required a laparoscopic resection in a young man. Sugarbaker *et al.* [11] in a published study reported an intraoperative rate of 49% for patients with hepatic metastasis undergoing cytoreduction.

## CONCLUSION

We present a rare case report of a subhepatic appendix and a concomitant interlobar hepatic bridge observed during a laparoscopic cholecystectomy, which we believe to be the first in the published literature.

## Data Availability

Data is available upon request.
